# Microwave axial dielectric properties of carbon fiber

**DOI:** 10.1038/srep14927

**Published:** 2015-10-19

**Authors:** Wen Hong, Peng Xiao, Heng Luo, Zhuan Li

**Affiliations:** 1 State Key Laboratory of Powder Metallurgy, Central South University, Changsha 410083, China

## Abstract

Randomly distributed carbon fibers (CFs) reinforced epoxy resin composites are prepared by the pouring method, the dielectric properties of CF composites with different fiber content and length have been performed in the frequency range from 8.2 to 12.4 GHz. The complex permittivity of the composite increases with the fiber length, which is attributed to the decrease of depolarization field, and increases with the volume fraction, which is attributed to the increase of polarization. A formula, based on the theory of Reynolds-Hugh, is proposed to calculate the effective permittivity of CF composites, and validated by the experiments. The proposed formula is further applied to derive the axial permittivity of CF and analyze the effect of fiber length on the axial permittivity.

Carbon fiber (CF), commercially available since the 1960s, has attracted worldwide interest as the reinforcement for the structural, electrode, electromagnetic shielding and absorbing materials^1^,^2^,^3^,^4^,^5^. Particularly, the microwave dielectric properties of composites filled with random CF have received a great deal of attention due to their high permittivity at low concentration and pronounced microwave dielectric dispersion^6^,^7^,^8^. However, the microwave absorbing performance has not been fully explored due to the lack of the anisotropic permittivity of CF. Owning to the difficulty in charactering CF, very few direct measurements of its anisotropic permittivity have been reported. Thus the dielectric mixture theory becomes the only available means to derive this basic data, which was developed as far back as 1891 when Maxwell tackled the problem of obtaining the effective electromagnetic properties of mixtures^9^. Since that time, many researchers have been pushing forward the theory^10^,^11^,^12^,^13^,^14^,^15^. And in recent years considerable attentions have been paid to develop the effective dielectric theory for CF composite. For example, Lagarkov *et al.* proposed a scale-dependent effective medium theory to calculate effective permittivity of stick composite^16^, and the method was proved to be accurate in predicting the permittivity of CF composite^17^, while others reported the equivalent circuit model was found useful in describing the effective dielectric constant of fiber composite^18^,^19^. More recently, linear regression analysis was used to derive isotropic permittivity of CF for the optimization of CF composite for microwave absorbing^20^. Furthermore, Balzano *et al.* applied Maxwell Garnett model to calculate the effective permittivity of CF mixture^21^, and in 2009 De Rosa *et al.* used Maxwell Garnett model to extract the isotropic permittivity of CF^22^. However, current theories suffer either from lack of solid physical ground by introducing fitting parameters or from disagreement with the CF microstructure by treating CF as isotropic material. Hence, the anisotropic permittivity of CF remains mysterious. Previously, we have derived the radial permittivity of CF^23^. Here we move next to extract the axial CF permittivity, thus the anisotropic permittivity of CF is completely obtained.

In this paper, we proposed a formula, based on the theory of Reynolds-Hugh, to calculate the effective permittivity of randomly distributed CF composite by neglecting fiber interaction at dilute concentration. The axial permittivity of CF with different fiber length is further derived with the help of proposed formula, and validated by experiments. Until now, no research with regards to derive axial CF permittivity was reported. Hence, our manuscript presents a competitive work.

## Results

### Measurement validity

**Figure 1:**
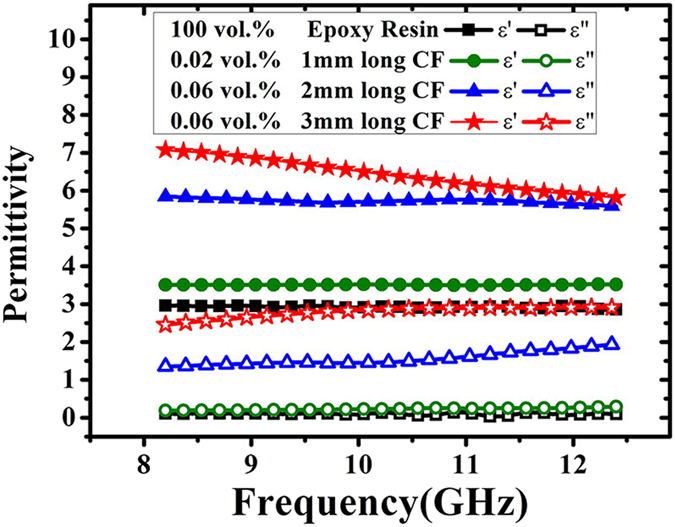
Frequency spectra of the complex permittivity of epoxy resin (solid and open black square lines), 1 mm long (solid and open green circle lines), 2 mm long (solid and open blue triangle lines) and 3 mm long (solid and open red star lines) CF/epoxy resin samples.

As expected, the permittivity is proportional to the density, the higher the density is, the higher the dielectric constant is^24^. In order to validate the effectiveness of the measured permittivity, it is necessary to fabricate samples with similar density. Due to the proper preparing process, little air bubble is generated within the resin matrix, the density of all CF/epoxy resin samples is around 1.1 g/cm^3^. The complex permittivity of the epoxy resin, 1 mm long (0.02 vol.%), 2 mm long (0.06 vol.%) and 3 mm long (0.06 vol.%) CF/epoxy resin samples are compared in Fig. 1.

The results of randomly distributed CF/epoxy resin composites are compared with epoxy resin sample for evaluating the validity of the measurements. First, the real and imaginary part of complex permittivity of the epoxy resin is around 2.95 and 0.1, which agrees well with the reported literature^23^,^25^. Second, the higher the volume fraction of CF is, the larger the permittivity is. Compared with that of 1 mm long CF sample, the permittivity of 2 and 3 mm long CF samples is relatively larger due to the higher filling fraction. The dielectric constant of 2 mm long CF samples is lower than that reported by Balzano *et al.*^21^, which is due to the resin matrix with smaller permittivity (2.95–0.1i). Finally, the permittivity of CF composites increases with fiber length, which agrees well with previous work^25^. Furthermore, theoretical research indicated agglomerate may bring larger permittivity^26^, thus the dielectric constant of CF composite with aggregation, which result from the preferred orientation of the fibers, is larger than that without agglomerate. Despite of epoxy resin with small permittivity, the measured permittivity of CF samples is still relative small, and the pouring method is believed to be effective in avoiding the preferred orientation of fiber^21^. Hence, the fibers here are believed to distribute randomly without preferred orientation.

### Modeling and validation

As indicated by the theory of Reynolds-Hugh, the effective electric field of the CF composite can be expressed as^27^:


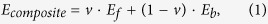


where 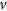 is the CF volume fraction, 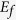 and 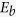 is the electric field inside the fiber and matrix. Without considering interaction between fibers, the polarization of CF composite is equal to the sum of polarization of fiber and matrix, which can be described as follows:


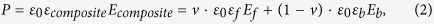


where 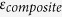 is the effective composite permittivity, 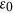, 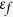 and 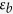 is the permittivity of the vacuum, CF and matrix respectively. After substituting Eq. (1) into Eq. (2), the effective permittivity of CF composite can be expressed as:


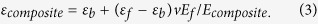


As indicated by Eq. (3), the lower the fiber filling fraction is, the lower the effective permittivity of fiber mixture is. Thus, compared with reported results^21^, the permittivity of CF sample with same fiber length is relative small. Moreover, the results show the permittivity of CF composites increases with fiber length. In order to analyze the effect of fiber length on the effective permittivity of randomly distributed CF composite, Eq. (3) can be expressed as:


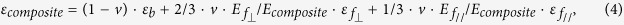


where 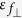
and 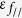
represents the radial and axial CF permittivity, 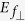
and 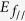
is the electric field inside fiber when the electrical field is perpendicular and parallel to the fiber axis, and 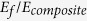 follows^23^:


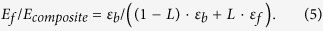


And the depolarization factor *L* along the electrical field is^18^:


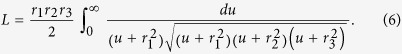


The Eq. (4) is valid in the hypothesis that the interaction between fibers is ignorable. And the interaction will increase with volume fraction in randomly distributed CF composite, especially around the percolation threshold^16^,^17^. Hence, the filling fraction of CF should be far lower than that of the percolation threshold for the proper use of the Eq. (4).

**Figure 2:**
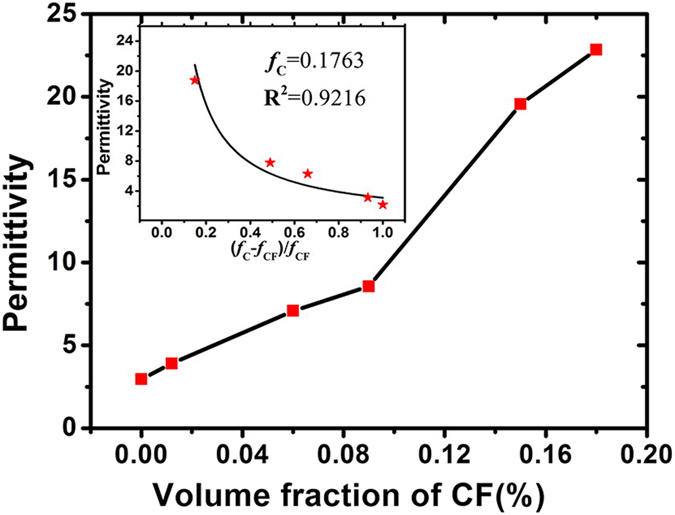
Variation of the dielectric constant of the3 mm-long CF/Epoxy resin composites on the volume fraction of CF at 8.2 GHz, and the inset in Fig. 2 shows the fit of the permittivity to Eq. (7).

As reported previously, the percolation threshold of fiber composite decreases with length-diameter ratio^16^. Thus, the longer the fiber length is, the lower the percolation threshold is. In order to assure the proper use of Eq. (4), it is necessary to check the percolation threshold of CF composites. However, due to the high electric insulation of epoxy resin, the electric conductivity of CF/epoxy resin composite is hard to be measured reliably, the measured results are easily affected by the location of the probe and measuring region, especially at dilute concentration. Fortunately, research shows the enhancement in the permittivity can also be used to determine the threshold volume fraction, the following power law is employed^7^.


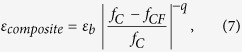


where 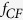 is the CF volume fraction,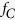 is the percolation threshold,
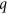
is a critical exponent of about 1. The permittivity of 3 mm long CF composite with different filling fraction at 8.2 GHz is compared in the Fig. 2, and the inset in Fig. 2 shows the fit of the permittivity to Eq. (7).

It is shown in the inset of Fig. 2, the experimental values of permittivity are in good agreement with Eq. (7), with the percolation threshold of 3 mm long CF composite 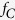
=0.1763 vol.% and coefficient of determination (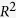
=0.9216). Thus the threshold volume fraction of 1 and 2 mm long CF composite is believed to be higher than 0.1763 vol.% due to the smaller length-diameter ratio. And the volume fraction of CF composite in Fig. 1 is equal to or below 0.06 vol.%, which is far less than the percolation threshold of 3 mm long CF composite. Thus, it is reasonable to ignore the fiber interaction at this dilute concentration. Then it is reliable to use Eq. (4) to derive the anisotropic permittivity of CF from the data in Fig. 1.

Previously, we have already derived the radial CF permittivity^23^. Here we move next to extract the axial CF permittivity, thus the anisotropy permittivity of CF is completely obtained. Referring to the Eq. (6), the depolarization factor 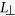 and 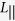 for 1 mm long CF with diameter of 7 μm are 0.49988 and 2.4313 × 10^−4^, respectively. The depolarization factor 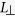 and 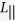 for 2 mm long CF are 0.49997 and 6.9274 × 10^−5^, and depolarization factor 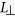 and 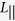 for 3 mm long CF are 0.49998 and 3.2996 × 10^−5^. After submitting Eq. (5) into Eq. (4), a formula, based on the theory of Reynolds-Hugh, is proposed for calculating effective permittivity of randomly distributed CF composite:


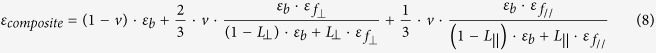


Substituting the CF filling fraction 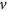, permittivity of composite 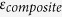, matrix 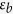 and the radial permittivity of CF
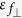
into Eq. (8), the axial CF permittivity 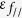
can be derived.

**Figure 3:**
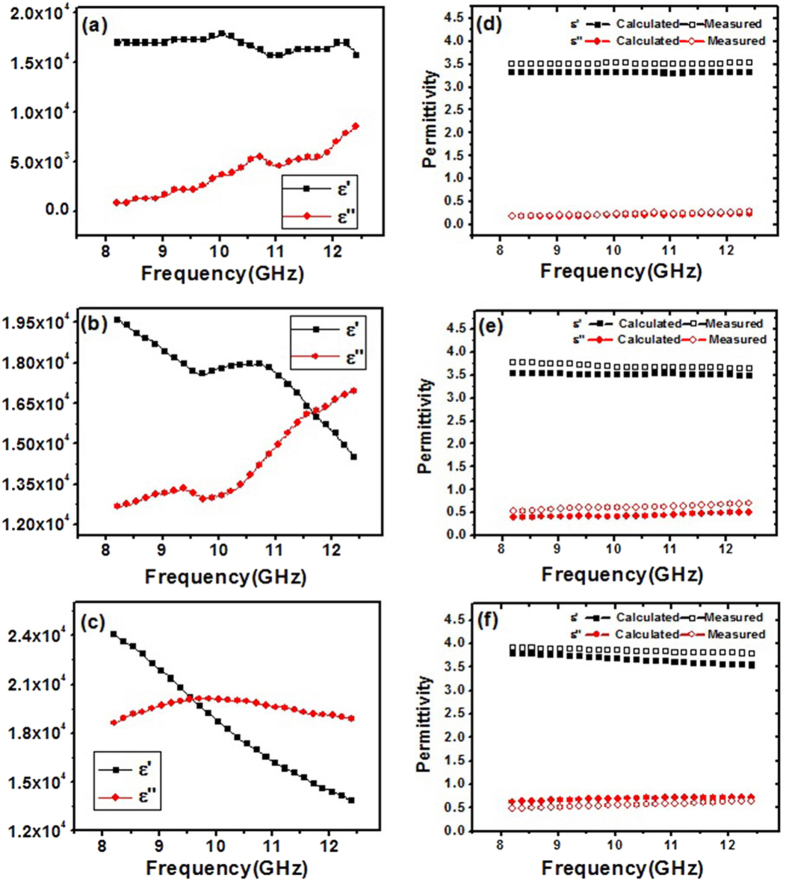
The axial permittivity of: (a) 1 mm long CF; (b) 2 mm long CF; (c) 3 mm long CF; comparison between calculated and measured permittivity of: (d) 0.01 vol.% 1 mm long CF composite; (e) 0.012 vol.% 2 mm long CF composite; (f) 0.012 vol.% 3 mm long CF composite.

The axial permittivity of 1, 2 and 3 mm long CF are derived by using data in Fig. 1, and are shown in the Fig. 3(a–c). The interaction is generally believed to decrease with fiber content, thus here we are to calculate the effective permittivity of CF composites with lower filling fraction. The accuracy of the proposed formula is checked by predicting effective permittivity of CF composite with different volume fraction. The comparison between calculated and measured permittivity of 0.02 vol.% (1mm long), 0.012 vol.% (2 mm long) and 0.012 vol.% (3 mm long) CF composite is shown in the Fig. 3(d–f), respectively.

As shown in the Fig. 3(d–f), the dielectric constant of randomly distributed CF composites with different volume fraction is well predicted with the derived axial permittivity, which means the proposed formula is suitable for deriving axial CF permittivity. And yet there still exists a slight difference between calculation and measurement, which may be caused by the weak interaction between fibers. As we know, the number N of the effectively interact fibers N ~ p (a/b)^2^, p is the fiber volume concentration, a and b is the length and radius of a fiber. For the composites considered here the number of the effectively interact fibers N ~ 10. For N≫1, the dispersion of the effective permittivity can happen even for the fibers that conductivity does not depend on the frequency. Hence, it can be found from Fig. 3(a–c), the dispersion of axial permittivity is more obvious with relative longer fiber. But, the hypothesis of neglecting the fiber interaction in Eq. (8) is still reasonable.

It should be noted that our method of deriving the axial CF permittivity is based on a specific DMT, in which fiber should be distributed randomly and homogeneously. And this DMT does not account for the fiber interaction. Previous researches indicated partial aggregation and inhomogeneity will exert influence on the composite permittivity^21^,^26^, and thus affects the obtained axial permittivity. Fortunately, the pouring method is believed to distribute fiber randomly and uniformly to avoid aggregation^21^. Meanwhile, the enhanced multipolar interaction resulted from increased aggregation will lead to the failure of DMT at high concentration^28^. Hence, we prepared CF composites with volume fraction as low as possible to weak the impact of interaction on the composite permittivity. Therefore, our results are reliable.

## Discussion

**Figure 4:**
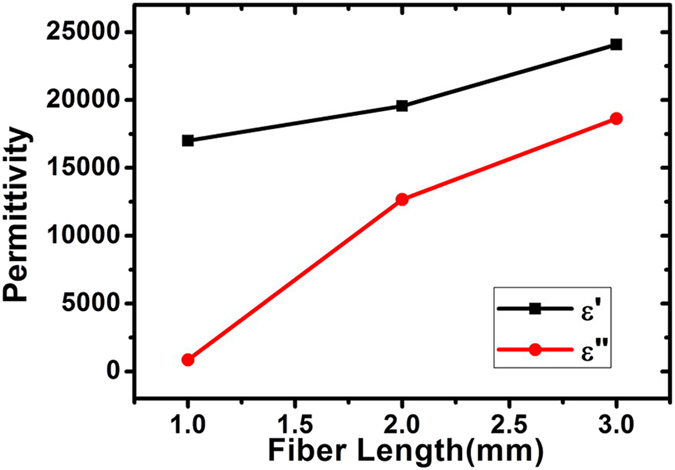
The relationship between fiber length and axial permittivity of CF at 8.2 GHz.

In order to analyze the effect of fiber length on the axial permittivity of CF, the axial permittivity of CF with different fiber length at 8.2 GHz is demonstrated in Fig. 4. The black square and red circle lines are the real and imaginary part of the axial permittivity.

Results show both real and imaginary parts of dielectric constant increase with increasing fiber length, which agrees well with trend predicted by an equivalent circuit^19^,^29^. Refer to the definition of the depolarization factor in Eq.(6), the larger the fiber length along the electrical field is, the smaller the depolarization factor L is. Due to the longer fiber length along the electric field, the depolarization factor 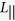 decreases from 2.4313 × 10^−4^ (1 mm long CF) to 3.2996 × 10^−5^ (3 mm long CF), thus both real and imaginary parts of axial permittivity of CF increase with fiber length. A greater increase is observed in the imaginary part of derived permittivity, which may be the deep reason for why long fiber always bring low percolation threshold^30^,^31^,^32^. It was generally believed the conductivity is proportional to the imaginary part of the permittivity, which follows: 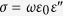, hence the CF composites with longer fiber length are more easy to exceed the percolation threshold. Moreover, much less fiber is needed to form a conductive network, and there is a greater probability of fiber to fiber contact with longer fibers. Therefore, the percolation threshold decreases with fiber length.

**Figure 5:**
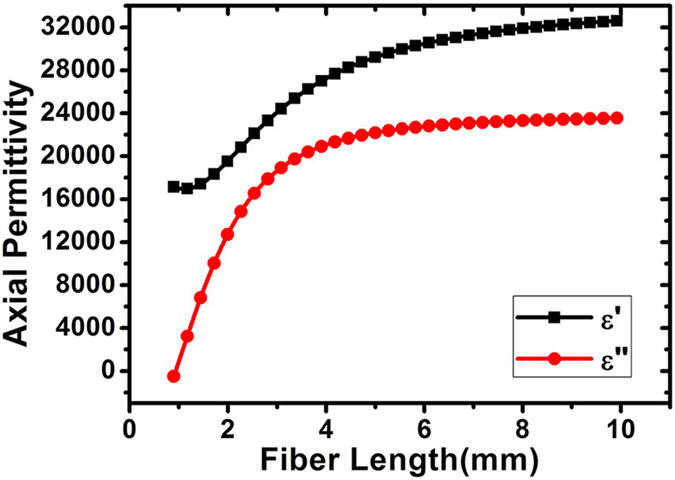
The effect of fiber length on axial permittivity at 8.2 GHz.

As shown in the Fig. 4, both real and imaginary parts of axial permittivity increase with fiber length, but to what extent does the axial permittivity become stable with increasing fiber length? A formula, previous used to analyze the effect of transversal section geometry on radial permittivity of CF, is proposed, which follows: 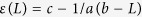
23, where a, b and c are fitting parameters, which are mainly dependent on the axial permittivity of fiber and matrix and inclusion geometry. According to Eq. (6), the depolarization factor 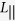 will decrease with fiber length. After fitting of data in Fig. 4 to the above formula, the relationship between axial permittivity and fiber length at 8.2 GHz is obtained and shown in the Fig. 5.

**Figure 6:**
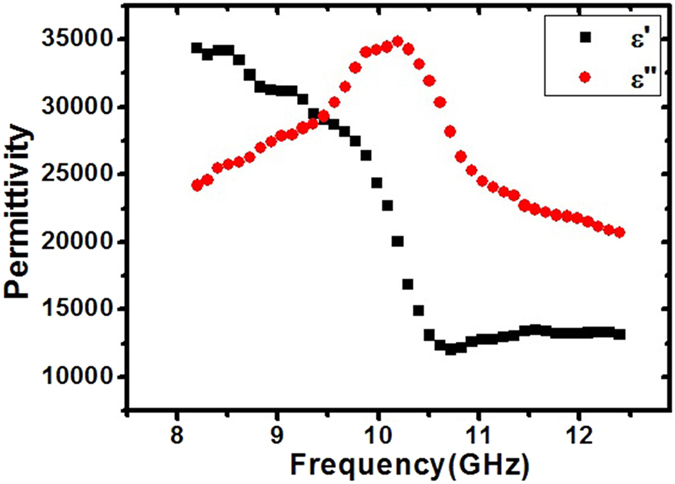
The axial permittivity of carbon fiber in X-band.

It is shown in the Fig. 5, the complex permittivity increases rapidly when fiber length is less than 5 mm, but after that the dielectric constant grows slightly. With the increase of fiber length, the decrease of depolarization factor becomes smaller, thus the increase of axial permittivity becomes smaller with the increase of fiber length. And the axial permittivity of CF 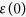 can be obtained by setting L as zero (shown in the Fig. 6).

According to the Transmission Line theory, the closer characteristic impedance of the material (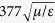) and free space (377) is, the better impedance match could be achieved, which means more electromagnetic wave could enter the material to be attenuated. As shown in the Fig. 6, the real part of axial permittivity of CF decreases with frequency, and the imaginary part of axial permittivity of CF peaks around 10.2 GHz, the dispersive behavior is close to the relaxation behavior given by the Debye theory^33^. As indicated by the Debye theory, the imaginary part of permittivity peaks at 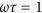, hence the relaxation time of CF here is 98.5 ps.

The real part of the axial permittivity is as high as above 10000, electromagnetic wave will be reflected directly due to the impedance mismatch at the air-dielectric interface. Compared with the radial permittivity^23^, the axial permittivity of CF is much smaller, which indicates CF exhibits strong dielectric anisotropy at microwave frequencies.

## Method

In order to characterize the microwave permittivity of randomly distributed CF/epoxy resin composites, it is important to prepare test samples with uniformly and randomly distributed fibers, as well as with suitable values of fiber content so that it is possible to perform correct measurement.

The epoxy matrix is prepared by mixing 87 parts of the epoxy resin (E44, Wuxi Resin Factory, China) with 13 parts of the flexibilizer (Dibutyl phthalate, Xilong Chemical Factory, China), and then the hardener (Ethylenediamine, Xilong Chemical Factory, China) is added at a concentration of 10:100 (by weight) to the mixture (epoxy resin and flexibilizer). The relative permittivity of the resin in X band is 2.95, the density is equal to 1.18 g/cm^3^.

The CFs used in this work are PAN-based T700 (Toray Industries Inc, Japan), whose tensile modulus is 294 GPa, d.c. electrical conductivity is 62500 S/m, and diameter is 6.8–7.2 μm.

The CF/epoxy resin composites are prepared by mixing the chopped CFs with the epoxy matrix before adding hardener. The composite is mixed until a uniform and homogeneous dispersion is achieved. Then the hardener is added. The test specimens are prepared by pouring the mixture into test flange (22.86 × 10.16 × 3 mm^3^), by which a better isotropy of the composite can be achieved^21^. After that, the samples are heated 12 h at 80 °C under normal pressure for the curing.

In order to measure the relative complex permittivity of CF/epoxy resin composites in the X-band, a vector network analyzer (VNA) (Agilent N5230A) is employed, and is carefully calibrated with thru-reflect-line (TRL) approach. The measurement accuracy of VNA is checked by pure epoxy resin sample. The dielectric constant is derived from the scattering (S) parameters with standard S parameter retrieval procedure. The density of the samples is measured using Archimedes method.

## Additional Information

**How to cite this article**: Hong, W. *et al.* Microwave axial dielectric properties of carbon fiber. *Sci. Rep.* **5**, 14927; doi: 10.1038/srep14927 (2015).
